# Stress-Induced Mutagenesis, Gambler Cells, and Stealth Targeting Antibiotic-Induced Evolution

**DOI:** 10.1128/mbio.01074-22

**Published:** 2022-06-06

**Authors:** John P. Pribis, Yin Zhai, P. J. Hastings, Susan M. Rosenberg

**Affiliations:** a Department of Molecular and Human Genetics, Baylor College of Medicine, Houston, Texas, USA; b Department of Biochemistry and Molecular Biology, Baylor College of Medicine, Houston, Texas, USA; c Department of Molecular Virology and Microbiology, Baylor College of Medicine, Houston, Texas, USA; d The Dan L. Duncan Comprehensive Cancer Center, Baylor College of Medicine, Houston, Texas, USA; e Interdepartmental Graduate Program in Integrative Molecular Biomedical Sciences, Baylor College of Medicine, Houston, Texas, USA; University of Toronto

**Keywords:** antibiotic resistance, antibiotics, cell subpopulations, evolvability, evolution, stress-induced mutagenesis, antievolvability drugs, drug resistance evolution

## Abstract

Mechanisms of evolution and evolution of antibiotic resistance are both fundamental and world health problems. Stress-induced mutagenesis defines mechanisms of mutagenesis upregulated by stress responses, which drive adaptation when cells are maladapted to their environments—when stressed. Work in mutagenesis induced by antibiotics had produced tantalizing clues but not coherent mechanisms. We review recent advances in antibiotic-induced mutagenesis that integrate how reactive oxygen species (ROS), the SOS and general stress responses, and multichromosome cells orchestrate a stress response-induced switch from high-fidelity to mutagenic repair of DNA breaks. Moreover, while sibling cells stay stable, a mutable “gambler” cell subpopulation is induced by differentially generated ROS, which signal the general stress response. We discuss other evolvable subpopulations and consider diverse evolution-promoting molecules as potential targets for drugs to slow evolution of antibiotic resistance, cross-resistance, and immune evasion. An FDA-approved drug exemplifies “stealth” evolution-slowing drugs that avoid selecting resistance to themselves or antibiotics.

## INTRODUCTION

Evolution drives real-world problems in infectious disease and biology, from dangerous new viral variants (1) to antibiotic resistance (2). An estimated 1.27 million deaths worldwide resulted from antibiotic-resistant infections in 2019 (3). The World Health Organization (WHO) has issued a call to action against evolution of antibiotic resistance in priority pathogens (4). Antibiotic resistance occurs either by transfer of resistance genes from one to another bacterium (reviewed in reference 5), or by *de novo* mutations that confer resistance. Although horizontal gene transfer (HGT) is important in many clinical circumstances (6, 7), for some specific widespread pathogens and widely used antibiotics, *de novo* mutations cause clinically relevant resistance.

*De novo* mutations can cause antibiotic resistance in various ways: they can alter the target protein and prevent antibiotic binding (8), or upregulate efflux pumps (9) or enzymes that degrade antibiotics (10, 11), reducing effective antibiotic concentrations. Mutations are the primary source of resistance of enterobacterial nosocomial infections in hospitals (12). In the World Health Organization (WHO) list of priority antibiotic-resistant pathogens (4), several acquire resistance by mutagenesis. These include Helicobacter pylori resistance to tetracycline (13), Mycobacterium tuberculosis resistance to isoniazid (14), and carbapenem resistance in *Enterobacteriaceae* (15). Mutagenesis is the main route to resistance to widely used fluoroquinolones (16) and the “last-chance” antibiotic daptomycin (17) and underlies chromosomally mediated colistin resistance (18). Moreover, even plasmid-borne β-lactamases, shared by HGT, require mutagenesis to confer resistance to newer-generation β-lactam antibiotics, which are then rendered ineffective (10). Identification of the mutagenic mechanisms that promote antibiotic resistance could allow new strategies to combat the now critical problem (4). The escape of pathogens from our immune defenses and drugs is a problem in the molecular, systems-biological, and populational mechanisms of evolution.

Mutations (including all *de novo* genomic changes) drive evolution, and our paradigm for both is changing. Mutagenesis and evolution are being recognized as dynamic, regulated processes with molecular mechanisms that can be both understood and, potentially, inhibited clinically (19–25). This view is necessitated by understanding of stress-induced mutagenesis: molecular mechanisms of mutagenesis that are upregulated by stress responses. The existence of stress-induced mutagenesis mechanisms implies that mutation rates, and the ability to evolve, increase preferentially when cells are poorly adapted to their environment, when stressed (reviewed in references 23, 25, and 26).

Stress-induced mutagenesis departs from ideas established before knowledge of the molecular basis of genes. Mutations were assumed to occur randomly both in time and in genomic space and constantly and gradually (27). Luria and Delbrück defined a mathematical relationship between the birth of mutations and cell divisions that occurred before exposure to a killing environment of lytic bacteriophage, in which Escherichia coli phage-resistant mutants were selected and then quantified (28). Because they used a killing selection for mutants, they saw only mutants already present and failed to detect any possible stress-induced mutagenesis.

Discovery of the bacterial SOS DNA damage response (29–32) led Harrison Echols to propose that stress sensing could, via the SOS response, promote genetic instability, and “inducible evolution” (33). The SOS response upregulates DNA damage tolerance and repair and instigates mutagenesis, prophage induction, and inhibition of cell division (reviewed in reference 34). Others argued, however, that SOS mutagenesis is an unavoidable by-product of repairing DNA damage, that nonmutagenic DNA repair could not evolve (e.g., see references 35 and 36), and that cells must repair DNA damage to survive.

The possibility of stress-inducible evolution (33) was difficult to consider until discoveries that mismatch repair, which corrects DNA replication errors, could be downregulated, increasing the mutation rate without assisting DNA repair (as shown in references [Bibr B37][Bibr B38][Bibr B40] and reviewed in references 23, 25, and 26), and that the general stress response was required for transposon movement (41, 42) and other mutagenesis under stress (43) and not for concurrent repair (44, 45). Many different stress responses are now documented to upregulate mechanisms of mutagenesis, including mutagenesis unrelated to the SOS response (e.g., see references [Bibr B46][Bibr B47][Bibr B52]). These various mechanisms promote aneuploidy (53, 54) (in eukaryotes), base substitutions and indels (insertions or deletions of one or a few base pairs) (reviewed here, and see references 25 and 26), transpositions (41, 42), and copy number alterations (CNAs) and other genome rearrangements (49, 50, 55). Additionally, reactive oxygen modifies transposase accuracy directly, without stress responses, and so, similarly, causes stress-induced transposon mutagenesis during oxidative stress (56).

In this review, we discuss current understanding of mechanisms of stress-induced mutagenesis. We examine mutagenesis induced by antibiotics and its promotion of antibiotic resistance and cross-resistance to antibiotics not yet encountered. We discuss two mechanisms of starvation stress-induced mutagenesis (21) that also underlie quinolone-induced mutagenesis (19, 57): two kinds of mutagenic repair of DNA breaks. Quinolones induce a switch from accurate to mutagenic modes of DNA break repair, using the general stress and SOS responses, which link mutagenesis to times of stress (57). The mutations are focused in hot spots near sites of DNA breakage (58) and occur in a transiently differentiated mutable “gambler” cell subpopulation (57). We examine how each of these departures from “random” mutagenesis can promote evolution. “Persisters” are subpopulations of transiently nongrowing or slowly growing cells that survive antibiotics temporarily, without a resistance mutation (reviewed in references 59 and 60) and cause relapse of infections by resuming growth after antibiotic clearance (59). We consider the possible relevance of persisters and other evolvable cell subpopulations to gambler cells and suggest criteria for choosing evolution-promoting molecules as potential targets for new drugs to slow evolution of antibiotic resistance and immune evasion, without selecting resistance to themselves or antibiotics. We call these “stealth” evolution inhibitors. In addition, we explore possible useful next steps.

## STRESS-INDUCED MUTAGENESIS IN BACTERIAL EVOLUTION

Most of this review is focused on mutagenesis and evolution in the laboratory. Stress-inducible mutagenesis, however, appears to contribute meaningfully to natural bacterial evolution. The large majority (more than 80%) of 787 E. coli natural isolates from diverse environments worldwide display stress-induced mutagenesis in a laboratory setting, showing that they possess the capability (46). Moreover, their ability to do so is correlated with the ecological niche of the isolates, suggesting that stress-inducible mutagenesis is selected (46). Selection of stress-induced mutagenic abilities is also supported by mathematical modeling. Bacterial populations capable of stress-induced mutagenesis showed improved fitness in changing environments (61–63). Separately, in whole-genome sequences of wild E. coli isolates, “mutational signatures” of the sequence differences between their genomes were dominated by specific base substitutions and indels that characterize mutagenesis that depends on the general (sigma-S, or σ^S^) stress response (64). The data imply that most natural variation arose by σ^S^-dependent (stress-induced) mutagenesis (64). Moreover, the multiple molecular mechanisms of stress-induced mutagenesis discovered in bacteria have predicted mechanisms at work in evolution of cancers (as reviewed in reference 25 and see references [Bibr B65][Bibr B66][Bibr B67]). Widespread evidence from bacteria to humans (25 [and see reference 68]) implies that much of the mutagenesis underlying evolution is stress induced.

## THE GENERAL STRESS RESPONSE SWITCH TO MUTAGENIC BREAK REPAIR

**FIG 1:**
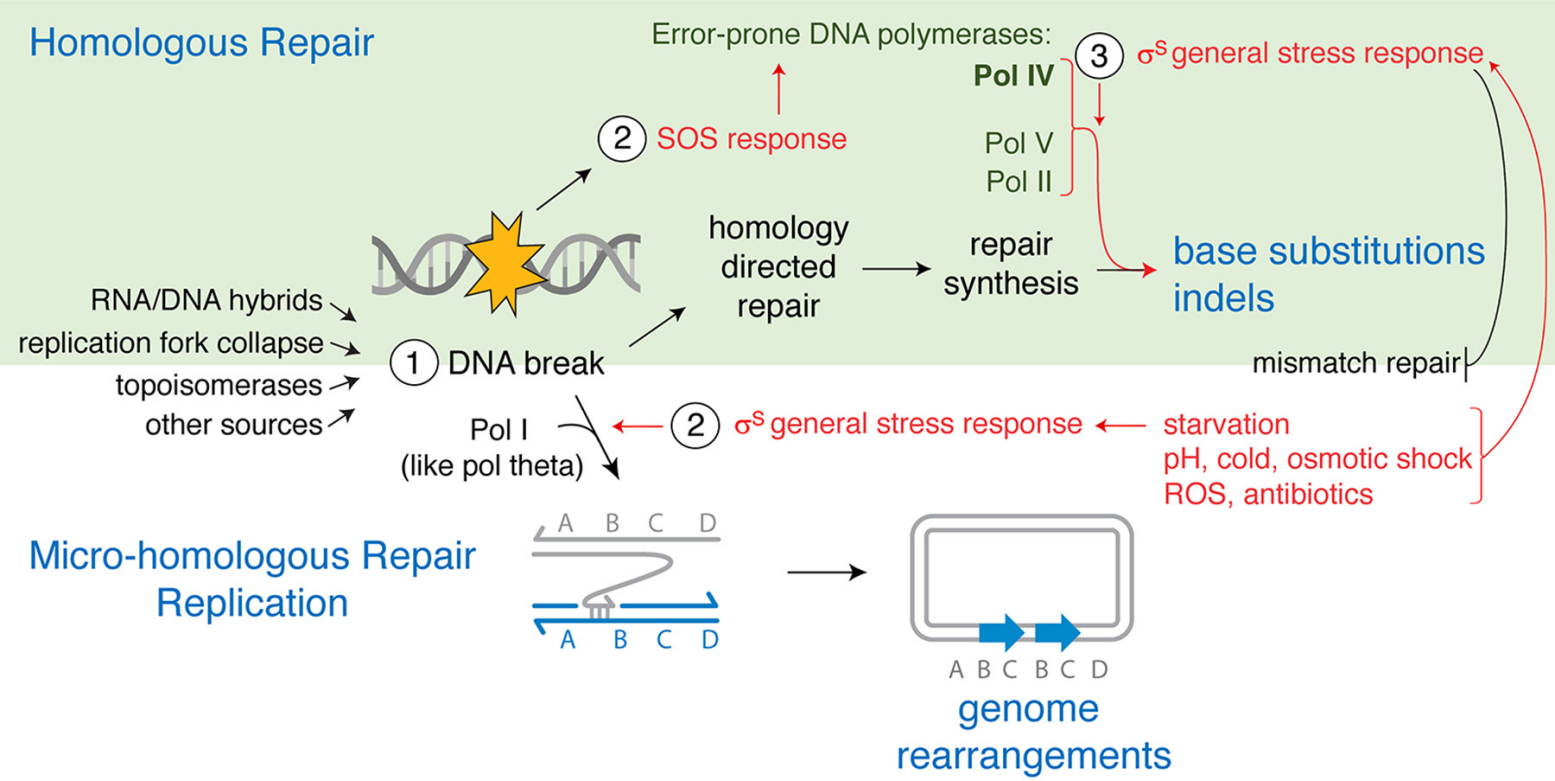
Temporal regulation of mutagenesis by stress responses in E. coli mutagenic break repair. (Step 1) DNA double-strand breaks (DSBs) are generated by various processes and can then be repaired by homologous or microhomologous repair mechanisms. (Top) During homology-directed DSB repair (HR) (reviewed in references 74 and 75), ssDNA exposed at the DSB ends base pairs with complementary sequence in a sister chromosome, promoting repair DNA synthesis. (Step 2) DSBs also induce the SOS response, which transcriptionally upregulates the error-prone DNA polymerases (Pols) IV, V, and II (82); however, repair remains accurate unless another stressor induces the general stress (σ^S^) response (44, 45). (Step 3) The σ^S^ response induces two kinds of switches to mutagenic DSB repair. In cells that are also SOS induced, the σ^S^ response, by unknown means, allows the use of, or persistence of errors made by, the error-prone DNA Pols in repair, causing base substitutions (45, 84) and indels (43, 44, 83, 166, 167). σ^S^ also downregulates mismatch repair (37–40, 88), which allows errors in DNA synthesis to persist. (Bottom) Less frequently, microhomologous MBR of a DSB occurs. It is SOS independent and requires (step 2) the σ^S^ response and DNA Pol I for template switching to regions containing microhomology (49, 50) (few complementary bases). The repair replication creates genome rearrangements. A duplicated chromosome segment is shown (blue arrows). Parallel lines represent base-paired DNA strands, and half arrowheads represent the 3′ DNA ends.

The general or starvation stress response in E. coli promotes at least two mechanisms of mutagenesis, both of which switch the otherwise accurate mechanism of DNA double-strand break (DSB) repair to mutagenic repair routes (Fig. 1). Both are activated by the general stress response, which occurs via production of the σ^S^ transcriptional activator (43–45) encoded by the *rpoS* gene (69). Bacterial sigma factors, including σ^S^, are interchangeable subunits of RNA polymerase (RNAP), which when plugged into RNAP, direct RNAP and transcription to some genes and away from others. The σ^S^ regulon, reviewed by Battesti et al. (69), is upregulated in response to starvation, cold shock, osmotic shock, acid shock, oxidative stress, and antibiotics (24, 70) and protects cellular hardware from damage during those stresses.

Mutagenic DNA break repair (MBR) in E. coli, studied during starvation, can occur during DSB repair by homologous recombination (44, 45, 58, 71–73), causing base substitutions and indels (25, 26) (Fig. 1, top). MBR requires three simultaneous events to occur (44, 45) (Fig. 1, top). First, a DSB must occur (Fig. 1, step 1) and be repaired by homology-directed repair (HR) (Fig. 1, top). DSB repair in E. coli uses the RecBCD homology-directed repair mechanism and occurs similarly in other bacteria (74, 75). DSBs are bound and “resected” by RecBCD, a DSB end-specific exonuclease that exposes single-stranded DNA (ssDNA) and then loads RecA onto the ssDNA end for repair (76). The RecA-DNA complex also signals DNA damage, which activates the SOS response (34, 77) (Fig. 1, top, step 2). The ssDNA-RecA complex displaces a DNA strand of similar sequence, usually in a sister chromosome, base pairs with the complementary sequence (reviewed in references 74, 75, and 78), and then initiates repair synthesis using the high-fidelity replicative DNA polymerase III (Pol III) (79). DSB repair and MBR require the RuvABC Holliday junction-resolvase complex (72–75) and RecA and RecBCD (44, 45, 71).

Second, the SOS response (Fig. 1, top, step 2) must be induced for homology-directed MBR (80), and this occurs following DNA breakage in about 25% of cells with a single reparable DSB (81). The SOS response transcriptionally upregulates about 40 genes in E. coli, including error-prone DNA Pols IV, V, and II (Fig. 1, top) (reviewed in references 34 and 82), all of which promote components of stress-induced mutagenesis. Pol IV is required for all homology-directed MBR and promotes formation of indels and base substitutions (44, 45, 83–85). The Pol IV 10-fold upregulation by the SOS response accounts for the SOS role in stress-induced MBR (86). Despite the 10-fold upregulation and efficient homology-directed DSB repair (44, 45), repair remains high fidelity and nonmutagenic unless a third event occurs: activation of the σ^S^ general stress response (Fig. 1, top, step 3) (43–45).

The σ^S^ response is an “AND gate” for MBR, for which at least two responses must occur simultaneously (SOS AND σ^S^). That is, the cell must sense at least two different stressors before committing to mutagenesis: DNA damage and the σ^S^ inducer. The σ^S^-inducing stressor most studied is starvation (21). Because a constitutive σ^S^ response allows MBR in the absence of any stress (44, 45), the stress response is required, but stress is not. The σ^S^ response, by unknown means, licenses the use of the SOS-upregulated DNA Pols in DSB repair and/or allows their errors to persist and become mutations (Fig. 1, top, base substitutions and indels). σ^S^ upregulates Pol IV about 2-fold (87), downregulates mismatch repair (38, 88), and may downregulate high-fidelity replicative DNA Pol III (about 2-fold reduction in mRNA) (89). Pol III competes with Pol IV in DSB repair-associated replication in cells (90) and biochemically at model strand displacement loops (D-loops) (91). Any of these effects could underlie the role of σ^S^ in stress-induced MBR.

Importantly, Pol IV and mutagenesis are not needed for efficient DSB repair (44, 45), which works as well (45) or better (44) in its absence, thus refuting arguments that high-fidelity DNA repair cannot evolve (35, 36). It did, but E. coli cells do not use it under σ^S^-inducing stress.

σ^S^ also promotes a “microhomologous” mechanism of DSB repair, which causes genome rearrangements (43) (Fig. 1, bottom), including copy number alterations (CNAs) and other rearrangements (49, 50, 55). Microhomologous MBR does not require an SOS response (83). Microhomologous MBR might occur in starving cells that lack a sister chromosome template for homology-directed repair or those not undergoing the SOS response upon DNA breakage. Microhomologous MBR (Fig. 1, bottom) occurs via a microhomology-mediated break-induced replication (MMBIR) mechanism (44, 50, 92, 93) and is reviewed elsewhere (25, 92).

## MUTATION HOT SPOTS AND CLUSTERS

MBR causes mutations near DSBs, implicating the tracts of DSB repair synthesis as the MBR sites (44, 58). When a site-specific DSB is delivered to the chromosome of starving cells, σ^S^- and Pol IV-dependent mutations occur maximally within the first kilobase pair on either side of the break site and fall off logarithmically to about 60 kb on either side of it, with a long tail of low-level Pol IV-dependent mutations up to a megabase pair away (58), presumably in the tracts of DSB repair synthesis. The mutations occur in clusters, with the probability of a mutation being higher at sites near another mutation (94). Mutation clusters can promote “concerted evolution” that requires multiple simultaneous mutations within a gene or linked genes to allow function. There is no evidence of increased DNA breakage during MBR in starving cells (93) compared with nonstressed cells (81, 95), only of the σ^S^-dependent increase in the mutagenicity of repair (44, 45). Thus, how and where spontaneous DNA breakage occurs might shape mutational landscapes and genome evolution. The first detailed maps of spontaneous DNA breakage in proliferating (mostly unstressed) E. coli cells show hot spots (96). Detailed maps of genomic mutations under various stress conditions would be invaluable for testing the prediction that these bacterial “fragile sites” (96) are mutable genomic regions.

## PROTEIN NETWORKS WITH STRESS RESPONSES AS HUBS

In a screen for MBR-defective mutants, our lab identified a network of more than 93 diverse proteins that promote MBR (21). A small number of these were most of the previously known MBR proteins: stress response activators, proteins that perform DSB repair, and error-prone DNA polymerases. However, most of the network proteins are highly diverse and were not obvious candidates for roles in mutagenesis. For example, the largest single category of network genes functions in the electron transfer chain (ETC). Functional tests showed that more than half of the 93 proteins promote mutagenesis, acting upstream of (i.e., before) activation of the three key stress response regulators in MBR: σ^S^ (31 proteins), σ^E^ (44 proteins), and the SOS response (6 proteins). For example, if the σ^S^ response is activated constitutively, the proteins required for its activation are no longer needed for MBR (21). Thus, most of the network proteins promote MBR by sensing stress and transducing the signals that activate the stress responses required to switch to error-prone DSB repair (21). Activation of stress responses appears to be the most important criterion for the E. coli decision to allow mutagenesis, having the largest allotment of genes. Moreover, the stress-response activators are nonredundant network hubs (21).

## QUINOLONE ANTIBIOTICS

In the rest of this article, we focus mostly on the very widely used fluoroquinolone antibiotics and how they induce mutagenesis to fluoroquinolone resistance and cross-resistance to other antibiotics, and we compare these findings with data on mutagenesis induced by other antibiotics, about which less is known.

Quinolone antibiotics bind and inhibit bacterial type II topoisomerases (topos) while they are in the act of relieving DNA supercoils (97), which result from unwinding of DNA during DNA replication and transcription. Type II topos bind DNA and cleave both strands, creating a DSB, and attach covalently to each 5′-end strand (98). The broken DNA allows another duplex to pass through, and is then religated, releasing the topo. The DSBs undo supercoils or allow decatenation of linked sister chromosomes following DNA replication (99). Quinolones bind type II topos after DNA breakage and before the religation step and so leave the DNA broken (97). The commonest route to resistance clinically (12) and in the lab (10, 100) is by *de novo* mutations that either alter the topo so that the drug no longer binds or cause upregulation of efflux pumps that export the drug.

## QUINOLONES INDUCE MUTAGENESIS AND ANTIBIOTIC CROSS-RESISTANCE

Ciprofloxacin (cipro) is the most commonly used fluoroquinolone (16, 101). “Subinhibitory” concentrations of cipro (below the minimal inhibitory concentration, or MIC) occur in ecosystems and during antibiotic therapies at the beginning, the end, and when doses are missed. Subinhibitory cipro can both induce and select cipro-resistant mutants (19, 100), making quantification of cipro induction of mutagenesis challenging. That fluoroquinolones induce mutagenesis was shown by exposing cells to another fluoroquinolone, norfloxacin, and then selecting and quantifying mutants resistant to antibiotics not yet encountered (102), antibiotic “cross-resistant” mutants. The norfloxacin-induced mutagenesis required reactive oxygen species (ROS) (102), which are induced by the antibiotic and also underlie its antibiotic (killing) activity (103). Yet, how the ROS might promote mutagenesis was unclear; direct oxidation of DNA bases seemed easy to imagine.

We found that cells grown in subinhibitory cipro at the “minimum antibiotic concentration (MAC),” at which the final CFU are 10% of identical drug-free cultures, induce mutations that confer resistance to two different antibiotics (57). Rifampin-resistant mutants carry base substitutions in the (essential) *rpoB* gene (104), and ampicillin-resistant mutants carry any loss-of-function mutation in the *ampD* gene (57, 105). These are induced about 30-fold and 15-fold, respectively, by MAC cipro (57). The cipro-induced rifampin- and ampicillin-resistant mutants have a slight growth disadvantage in MAC cipro and so are not selected by the cipro. Rather, *bona fide* induction of mutagenesis occurs (57). Base substitutions and indels are generated along with larger genomic rearrangements, including deletions in the *ampD* gene (57). In Fig. 1 (bottom), the microhomologous rearrangement pathway is shown and requires the σ^S^ and not the SOS response (83), and it might account for the cipro-induced larger deletions (92). The starvation stress-induced MBR mechanism (Fig. 1, top) provided a useful entry into how cipro induces mutagenesis (57).

### Cipro-induced mutagenesis is MBR.

Cipro-induced mutagenesis occurs via the stress-induced MBR pathway, requiring proteins of DSB repair, the stress-response regulators for the SOS and σ^S^ responses, and the error-prone DNA polymerases IV, V, and II (57) (Fig. 1, top). Supporting an MBR mechanism, cipro-induced mutagenesis is blocked by an induced DSB end-specific binding protein, Gam of phage Mu (57), demonstrating a role for DSBs. Cipro-induced mutagenesis also requires ROS and is blocked by ROS-quenching agents and an inhibitor of Fenton chemistry (57), which generates ROS. Cipro-induced DSBs, quantified as foci of GamGFP (a fusion of the phage Mu Gam protein to green fluorescent protein) (95), are unaffected by ROS-reducing agents (57), indicating that the role of ROS in MBR is not generating the DNA breaks. As described below, ROS promote mutagenesis by activating the σ^S^ general stress response.

### Mutable gambler cell subpopulation via general stress response.

**FIG 2:**
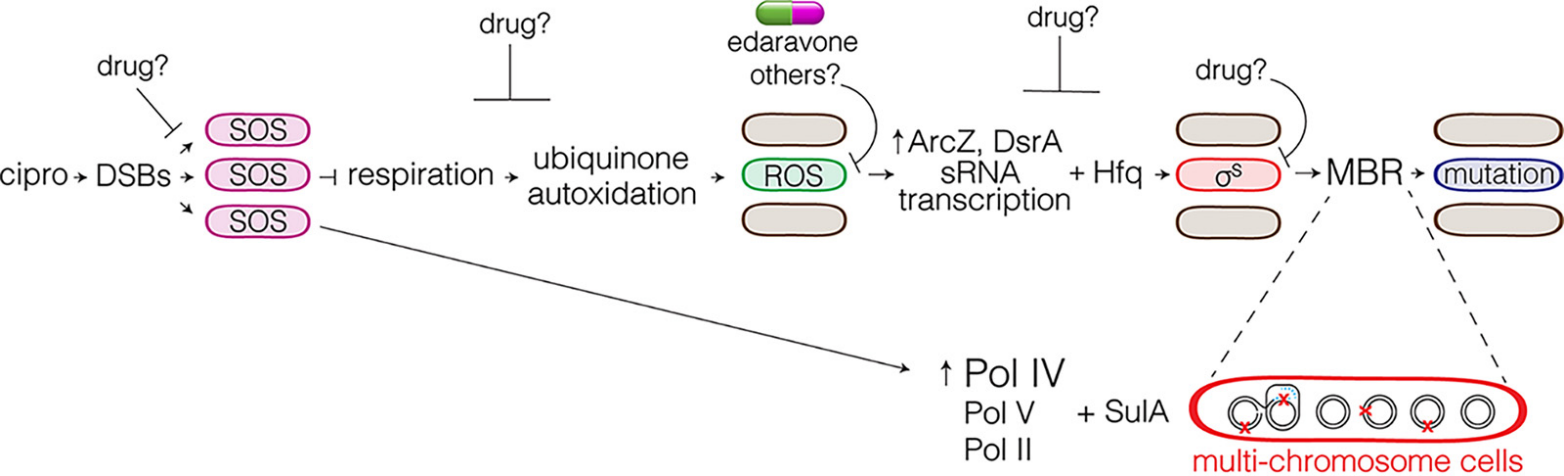
Pathway to and potential intervention points in formation of mutable gambler cells. cipro-induced DSBs activate the SOS response, which slows aerobic respiration (140). We suggest that increased autoxidation of reduced ubiquinone leads to (as observed in reference 57) a cell subpopulation with high levels of reactive oxygen species (ROS). ROS activate σ^S^ by upregulating transcription of sRNAs DsrA and ArcZ (57), which, with the Hfq RNA chaperone, increase translation of *rpoS* (σ^S^) mRNA in the cells with high ROS (57). This σ^S^-high “gambler” cell subpopulation allows mutagenic DNA break repair (MBR) (Fig. 1, top) and produces antibiotic cross-resistant mutants induced by cipro (57). The gambler subpopulation is transiently mutable (57). The SOS response also upregulates the SulA inhibitor of cell division, which promotes formation of multichromosome cells (168), which facilitate cipro-induced MBR (57). Potential antievolvability drug targets (depicted as “–| target,” in which “–|” indicates inhibition) have been identified by the discovery of the various steps of gambler cell formation and the action illustrated. The FDA-approved human drug edaravone inhibits gambler cell formation by quenching ROS (57), which does not select antievolvability drug resistance. Previous and proposed drugs to target the SOS response (155–157), DSB repair (158), any other DNA repair (154) and/or the error-prone DNA polymerases do reduce fitness in the antibiotic and so select resistance.

Antibiotics, including fluoroquinolones, induced the SOS (106–108) and general stress responses (24, 70) in studies that used bulk cell measurements. At the single-cell level, an interesting transient “differentiation” is seen. Flow cytometry and microscopy with ROS stain and fluorescence reporter genes for an active SOS (81, 109) or σ^S^ response (21) revealed the cascade of events outlined in Fig. 2 (57).

Cipro-induced DNA breaks were visible as GamGFP foci (95) in essentially all cells, as was the SOS response (57) (Fig. 2). The SOS response and DSBs are unaffected by ROS quenching, indicating that the DSBs and SOS response occur independently of ROS (57). Intriguingly, the sequences of the cipro-induced mutations do not show the ROS-mediated mutation signature of 8-oxo-dG (oxidized guanine: G·C→T·A and A·T→C·G) (110), reinforcing the conclusion that the ROS role in mutagenesis is not via oxidized (damaged) DNA (57).

Surprisingly, ROS-dyed and σ^S^-active cells composed only a roughly 20% cell subpopulation. The same cells display first ROS and then σ^S^ activity. The ROS induce the σ^S^ response, in that quenching the ROS prevented σ^S^ induction (Fig. 2) (57). As indicated in Fig. 2, ROS induce transcription of two small RNAs (sRNAs), ArcZ and DsrA, which with their RNA chaperone Hfq (57) increase translation of *rpoS* mRNA (69) (Fig. 2). Removal of any of these components prevented σ^S^ induction and mutagenesis (57) (Fig. 2). Additionally, the ROS ceased to be needed for mutagenesis if the σ^S^ response was artificially upregulated (57). Thus, the major role of ROS in cipro-induced mutagenesis is inducing the σ^S^ response, which subpopulation cells do by upregulating the two sRNAs (Fig. 2). This is unlike ROS roles in starvation stress-induced MBR (111), DNA damage, and antibiotic activity (112, 113).

The σ^S^-active cells, enriched by fluorescence-activated cell sorting (FACS), generated most of the cipro-induced mutants—more than 400 times more than arose from the σ^S^-inactive cells (57). Thus, ROS activation of σ^S^ creates a mutable “gambler” cell subpopulation. The gamblers “experiment” with mutagenesis, which might lead to either adaptation or loss of fitness, while most of the cells take no similar risk (57) (Fig. 2). This might be a “bet-hedging” strategy (114, 115), in which some members of a population are more likely to succeed by adaptation to a stressful environment and others stay the course, and so benefit if the environment reverts to its prestress state.

Gamblers are transient in that the mutants they generate do not retain a σ^S^-activated phenotype (57). Whereas low mutation rates under stressful conditions can limit adaptability (62, 63), populations of constitutively hypermutating cells show reduced long-term fitness (116). The transient gambler subpopulation appears to be an intermediate in which short-term increases of mutability may promote adaptation under stress without harming long-term fitness once adaptation occurs.

In one mathematical model, the apparent mutability of gamblers might have resulted from differential cell death (117); however, death rates were equal in gamblers and nongamblers, ruling out this hypothesis (57). This apparent discrepancy might reflect the model’s assumptions of no antibiotic-induced increase in chromosomes per cell (discussed below) and that cells in a population are equally mutable (117). The gambler subpopulation shows neither to be the case (57).

Many antibiotics activate σ^S^ (24 [reviewed in reference 23]), suggesting that MBR may be a conserved response for survival of antibiotics (70). Whether other antibiotics or σ^S^ inducers differentiate transient mutable gambler cell subpopulations and MBR remains to be determined. In starving colonies, cell subpopulations have been observed that have stress responses activated (118) and might be gamblers.

## EVOLVABLE CELL SUBPOPULATIONS, PERSISTERS, AND METABOLISM

Transient cell subpopulations provide alternative physiological options that allow survival, with or without mutation(s) or horizontal gene acquisition. Bacillus subtilis activates both starvation stress-induced mutagenesis and a subpopulation “competent” for DNA uptake with the same ComK transcriptional activator (119). The mutants are not enriched among transformants (52), suggesting alternative responses to the same stress. Plasmid transmission (HGT) and loss may also affect antibiotic resistance, with high conjugation frequency (10^−3^) promoting plasmid transmission and the resistance conferred and high loss (10^−3^) causing plasmid eradication and loss of resistance (120).

Present at about 10^−5^ of the population, persisters survive β-lactams (121, 122), fluoroquinolones (121–124), and aminoglycosides (121, 122) and underlie much of antibiotic treatment failure (reviewed in references 60 and 125). Persisters can form stochastically (126) or be induced via stress responses, including SOS (123) and σ^S^ (127), similarly to gamblers (57). Persistence resembles antibiotic “tolerance” (reviewed in reference 60), a physiological state in which whole populations survive even higher antibiotic levels (60) without a mutation.

Whereas persisters are found under high doses (60), gamblers and mutagenesis are maximal with “subinhibitory” antibiotics (19, 24, 57, 102) (10% survival). Gamblers might become or harbor future persisters or could be an alternative or unrelated program.

Reduced energy metabolism characterizes gamblers and allows persisters to withstand most antibiotics. For example, tricarboxylic acid (TCA) enzyme promoter activity varies in proliferating cells, and those with low TCA gene transcription become persisters (121). Some antibiotics kill regardless of metabolic activity, but are toxic at high doses. Zheng et al. (122) combined these with antibiotics that attack metabolically active cells to eradicate persisters (122). The metabolism-dependent antibiotics kill most of the cells, while rare persisters succumb to the metabolism-insensitive killers at lower, nontoxic doses (122).

Metabolism can affect resistance directly. Mutations that reduce TCA cycle activity cause resistance to some antibiotics (128) and appear in many clinically relevant pathogens (128). Similarly, the electron transfer component ubiquinone (UQ) acts early in gambler cell differentiation, upstream of reactive oxygen (57) (Fig. 2), which may result from ubiquinone autooxidation (129, 130) (discussed in the next section). Even “taking the chance” that some cells become mutable begins only when metabolism is threatened. Matic and colleagues suggest that energy metabolism is a key universal sensor for many stresses, including antibiotics (70).

In growing cells, spontaneous mutations, seen as foci of MutL-GFP mismatch-interacting protein (131), occurred mostly in subpopulations with stress responses activated (132), detected with fluorescence reporter genes. Cells with high SOS, RpoH/σ^H^ heat shock protein (protein stress), or OxyR oxidative stress response activity showed more MutL-GFP foci than those without stress response induction, linking spontaneous mutability to stress and stress responses (132). Furthermore, the cells with increased mutations also showed increased translation errors (132), suggesting a vicious cycle of mutations fueling poor proteostasis, which because proteins make DNA, feeds back to increased mutability. Rarely examined, translation errors might often accompany mutagenesis.

### Do efflux pumps induce gamblers?

Surprisingly, like gamblers, subpopulations with high activity of the AcrAB-TolC efflux pump (133, 134) show reduced levels of MutS mismatch repair protein and an increased mutation rate (135). Deletion of *acrB* blocked pump activity and MutS reduction. We hypothesize that increased efflux activity reduces the effective within-cell drug concentration to subinhibitory levels that both aid survival and induce mutagenesis (57, 102): a one-two punch against the antibiotic. The NorA efflux pump promotes both immediate survival and the evolution of cipro resistance in Staphylococcus aureus (136), and its chemical inhibition by reserpine reduced cipro resistance, supporting the hypothesis that preventing cipro efflux can raise the in-cell drug concentration (136) to beyond the subinhibitory level at which mutagenesis is induced (57, 102).

Most evolutionary interpretations (35, 36) and models (117) assume homogeneous populations. The variability of metabolic rates, prevalence of drug-induced and spontaneous mutations and persisters in metabolically depressed subpopulations, and gambler promotion of adaptability (55, 57, 135, 137–139) indicate that those models could be improved by incorporating heterogeneity. Evolvable cell subpopulations may be the rule, not the exception.

### Why some cells and not others? First steps.

How mutable subpopulations begin can reveal circumstances that necessitate acceleration of evolution. For gamblers, the ROS-high cell subpopulation (and mutagenesis) (Fig. 2) are induced by an SOS response in all cells (Fig. 2), which is dependent on UbiD synthesis of ubiquinone (UQ), a component of the electron transfer chain (ETC). UQ was not needed if σ^S^ was artificially upregulated, indicating that subpopulation induction is its sole role in MBR. The SOS response could promote ROS by its suppression of aerobic respiration (140), which causes autoxidation of reduced quinols, leading to ROS (129, 130). There might be heterogeneity in the SOS slowing of the ETC and a respiration threshold below which autoxidation of ubiquinone occurs. ETC activity might vary between cells, or, alternatively, heterogeneous production of an SOS-regulated protein(s) might cause only some cells to slow the ETC. SOS-induced TisB and DinQ disrupt membrane potential (124, 141) and so are candidates for an ETC inhibitor. The distribution of their induction among cells is unknown (142). Coupling mutagenesis to the ETC (21, 57) highlights ATP production in basic sensing of stress.

### Multiple chromosomes and mutagenesis.

E. coli cells grown in fluoroquinolones form long, multichromosome cell “filaments” (143). The SOS-induced inhibitor of cell division SulA (144) blocks polymerization of the microtubule-like cell division (FtsZ) ring, causing more chromosomes per single long cell “filament” (145, 146) (Fig. 2, bottom). SulA (144) promotes mutability in both starved (80) and cipro-treated cells, both per cell and per chromosome (57). The mutability might reflect a requirement for complementation of deleterious mutations long enough to make an adaptive mutation. Recombination between chromosomes might be the advantage (57, 143) for generating the mutants by MBR and/or for losing or buffering deleterious alleles. Mathematical modeling indicates that multichromosome cells survive increased mutation rates better than nonfilaments (57). Cipro-induced mutability shows both heterogeneous mutability between cells and promotion of mutations in multichromosome cells (Fig. 2), both evolution accelerators as predicted by modeling (57, 63).

In a striking similarity to gamblers (57), multiple chromosomes promoted persister formation directly (147). Cells with more than 1 chromosome survived quinolones better than single-chromosome cells separated by FACS (147). Moreover, the survival was RecA and RecB dependent, supporting the need for recombinational repair and/or for an SOS response to survive cipro-induced DSBs (19).

## TARGETS FOR ANTIEVOLVABILITY DRUGS

Mechanisms of stress-induced mutagenesis promise to reveal possible targets for “antievolvability” drugs to slow evolution of antibiotic resistance, cross-resistance, and immune evasion (19, 21, 22, 25) for better clinical outcomes (19–22, 24, 25). With identification of mechanisms and molecules that promote evolvability, it may be appropriate to consider criteria for choosing antievolvability drug targets. One approach could be to target essentially any protein required for stress-induced mutagenesis (or other evolution), preferably in various bacteria and antibiotics. A more deliberate “stealth” approach could focus, we suggest, on targets the loss of which causes no immediate fitness decrease—as for example, the loss of DNA repair proteins does—so that resistance to the antievolvability drug will not be selected directly. In this light, some previously proposed (Mfd) or targeted (RecA, SOS) evolvability-promoting molecules include nonstealthy targets that select resistance.

### Targeting proteins also needed for antibiotic survival.

Mfd is an RNAP translocase that functions in transcription-coupled nucleotide excision repair (NER) (TCR), among other roles. Mfd promotes mutagenesis (93, 148–151) to drug resistance (93, 150) and was suggested as a possible target for antievolvability drugs because it acts in diverse bacteria, including in the E. coli MBR mechanism (93). In E. coli MBR, Mfd promotes DNA DSBs at some genomic sites (93) in a pathway that also required RNA-DNA hybrids and the σ^E^ membrane protein stress response regulator (93, 152). An enzyme-induced DSB delivered near the mutation reporter gene substituted for all of these components, indicating their roles in generation of the spontaneous DSBs (93, 152). Mfd was postulated to promote DSB formation and mutagenesis by stabilizing RNA-DNA hybrid “R-loops” in DNA, which can prime DNA synthesis/replication that creates a DSB when it encounters a single-stranded nick in the DNA (93). σ^E^-dependent transcription was postulated to generate the RNA in the R-loops (93, 152).

Mfd also promotes mutagenesis in Bacillus subtilis (148–150, 153), Pseudomonas aeruginosa, and Salmonella enterica serovar Typhimurium, including within host cells (150). In B. subtilis, ComK-dependent (52) stress-induced mutagenesis requires Mfd and UvrA, a nucleotide excision repair (NER) protein that works in TCR and global (Mfd-independent) NER, implying that mutagenesis occurred dependently on TCR (148, 149); possible molecular mechanisms have not been defined. In another B. subtilis mutagenesis assay (150), Mfd-mediated mutagenesis also required UvrA, transcription, and Mfd interaction with an RNA polymerase subunit, implicating TCR (150). The authors suggest targeting Mfd or its interaction with the RNA polymerase to inhibit mutagenesis and antibiotic resistance (154).

### “Stealth” targeting of network hubs not needed for immediate antibiotic survival.

Another possible approach to targeting evolution makes use of a functional network analysis of evolution-promoting mechanisms. Functional network analysis of starvation stress-induced MBR showed the stress response activators to be key nonredundant hubs in the MBR network (21), making them attractive targets (21), inhibition of which might collapse the entire network. These hubs include σ^S^ and SOS response activators (21). Current inhibitors of the SOS response target RecA (activator), LexA (155–157) (repressor), and RecB/AddAB (158) (activators). Inhibition of these proteins increases bacterial killing (by blocking DNA repair) and so may be expected to select strongly for resistance (19): a nonstealth approach. We suggest aiming new drugs, instead, at evolution-promoting hubs that have little effect on survival of antibiotics. In the pathogen Candida albicans, HSP90 inhibitors (159) and the natural product beauvericin (160) prevent the evolution of resistance to antifungal drugs without altering killing. Resistance to beauvericin and similar evolvability inhibitors is, therefore, unlikely to be selected directly.

As a proof of concept, the ROS-reducing FDA-approved drug edaravone inhibits formation of cipro-induced gambler cells and mutagenesis (57) (Fig. 2). Edaravone is used for amyotrophic lateral sclerosis (ALS) and cerebral infarction (161). Edaravone did not change cipro induction of DSBs, the SOS response, or antibiotic killing (Fig. 2) (57), any of which could select edaravone- and cipro-resistant cells. Antioxidants, including edaravone, given either with antibiotics or alone, were beneficial in mouse infection models (162–164). The authors studied immune-modulating effects and not antibiotic resistance, a potential factor in their effectiveness. In humans, edaravone reduced septic shock and mortality in septic peritonitis patients receiving standard care (including antibiotics) (165). This might result partly from slowed evolution of antibiotic resistance; however, data on resistance were not reported. Edaravone is a promising proof of concept for stealth drugs that decrease evolvability without selection for antibiotic resistance (or edaravone resistance) because it reduces mutagenesis, not survival (57). Other possible hub-related targets for antievolvability drugs in cipro-induced MBR are noted in Fig. 2 (not meant to be an exhaustive list). Future analyses of evolution-promoting functional networks may reveal other promising targets as network hubs.

Antievolvability drugs might be used as adjuvants to traditional antibiotics, to extend their utility by slowing development of resistance (19, 22, 25, 57). Alternatively, as monotherapies, the slowing of pathogen evolution might tilt their evolutionary races against our immune defenses in favor of the immune system and allow clearance of infections without reducing the beneficial diversity of our microbiota with antibiotics (21, 22, 57).

## CONCLUSIONS

Mutagenesis mechanisms upregulated by stress responses promote mutagenesis preferentially when cells are maladapted to their environment and speed adaption in stressful and changing environments (models shown in references 62 and 63). In stress-induced mutagenic break repair, upregulation of mutagenesis does not aid cell survival of the DNA breaks (44, 45)—but may be induced because it accelerates adaptation (62, 63) when cells are stressed and struggling to survive. Gambler cells depart further from random mutagenesis, honing regulation further by limiting the risks of mutagenesis to part of a population that experiments with new genotypes, while other parts “hedge” the population’s “bets” by remaining stable. An antimutagenic FDA-approved drug, edaravone, quenches ROS and prevents formation of the gambler cell subpopulation in the laboratory (57) (Fig. 2): an example of “stealth drugging” evolution without selecting resistance directly. Edaravone might or might not be optimal for clinical inhibition of antibiotic resistance. There are likely to be more, as-yet-undescribed regulatory steps in quinolone-induced mutagenesis and other antibiotic-induced evolvability mechanisms, some of which might afford attractive potential drug targets. Understanding the details of the mechanisms of mutagenesis is likely to expand options for combating the evolution of pathogens and antibiotic resistance.
